# Supplementary material for: Galectin-8 modulates human osteoclast activity partly through isoform-specific interactions
Source: Life Sci Alliance. 2024 Feb 23;7(5):e202302348. doi: 10.26508/lsa.202302348 (PMC10895193; doi:10.26508/lsa.202302348)
Supplement: Supplementary file 1 [file LSA-2023-02348_TableS1.docx]

**Supplemental Table S1.** Interactome of the short and long galectin-8 isoforms

| **Prey** | **SAINTexpress Score** | | **Interactor** | **GO: BP** | **References** |
| --- | --- | --- | --- | --- | --- |
|  | **LGALS8L** | **LGALS8S** |  |  |  |
| ABCC1 | 1.0 | 1.0 | Shared | Vascular process in circulatory system; Cellular response to peptide; Vascular transport; Transport across blood-brain barrier; Organic molecule transport; Response to amyloid-beta; Vascular transport | 15; 16; 17 |
| ABCC4 | 1.0 | 0.87 | Shared | Vascular process in circulatory system; Vascular transport; Transport across blood-brain barrier; Organic molecule transport; Platelet degranulation; Vascular transport | 15; 16; 17 |
| **APP** | 1.0 | 0.97 | Shared | Regulation of synaptic transmission; Cellular response to peptide; Organization of extracellular structure components; Spontaneous synaptic transmission; Cognitive activity; Response to amyloid-beta; Cell junction assembly; Platelet degranulation; Neuron recognition; Cell recognition | --- |
| ATG9A | 0.99 | 1.0 | Shared | --- | 15; 16; 17 |
| BSG | 0.77 | 0.96 | Shared | Cell recognition; Organization of extracellular structure components; Dendrite self-avoidance; Virus host interaction; Cellular adhesion via plasma membrane adhesion molecules; Neuron recognition; Symbiont host interaction | 8; 10 |
| **CADM1** | 0.97 | 1.0 | Shared | Cell recognition; Cellular adhesion via plasma membrane adhesion molecules | --- |
| CD320 | 0.81 | 0.97 | Shared | --- | --- |
| **CLCN3** | 1.0 | 1.0 | Shared | --- | 15; 16; 17 |
| **CLCN7*** | 0.0 | 0.74 | Specific to LGALS8.Short | --- | 15; 16; 17 |
| CPD | 1.0 | 1.0 | Shared | --- | --- |
| ECE1 | 0.99 | 1.0 | Shared | Vascular process in circulatory system | 15; 16; 17 |
| **ITGA4** | 0.0 | 1.0 | Specific to LGALS8.Short | Cellular response to peptide; Organization of extracellular structure components; Activities mediated by integrin; Heterotypic cell-cell adhesion; Import into the cell across plasma membrane; Response to amyloid-beta | 13; 15; 16; 17 |
| **ITGA5** | 0.5 | 1.0 | Specific to LGALS8.Short | Organization of extracellular structure components; Negative regulation of anoikis; CD40 signaling pathway; Virus host interaction; Activities mediated by integrin; Cognitive activity; Heterotypic cell-cell adhesion; Cellular adhesion via plasma membrane adhesion molecules; Cell junction assembly; Symbiont host interaction | 3; 4; 5; 11; 13; 14; 15; 16; 17 |

*Not included in Figure 7-B; Gene in bold are related to osteoclast biology

**Supplemental Table S1. Continued**

| **Prey** | **SAINTexpress Score** | | **Interactor** | **GO: BP** | **References** |
| --- | --- | --- | --- | --- | --- |
|  | **LGALS8L** | **LGALS8S** |  |  |  |
| **ITGB1** | 1.0 | 1.0 | Shared | Regulation of synaptic transmission; Organization of extracellular structure components; Negative regulation of anoikis; Organic molecule transport; CD40 signaling pathway; Spontaneous synaptic transmission; Virus host interaction; Activities mediated by integrin; Heterotypic cell-cell adhesion; Import into the cell across plasma membrane; Cellular adhesion via plasma membrane adhesion molecules; Symbiont host interaction | 2; 3; 4; 5; 6; 7; 9; 11; 12; 13; 14; 15; 16; 17 |
| **LAMP1** | 0.0 | 0.99 | Specific to LGALS8.Short | Virus host interaction; Symbiont host interaction | 1; 10; 15 |
| **LAMP2** | 1.0 | 1.0 | Shared | Platelet degranulation | 1; 15 |
| LGALS3BP | 0.96 | 0.95 | Shared | Platelet degranulation | 10; 15 |
| LNPEP | 1.0 | 1.0 | Shared | --- | 15; 16; 17 |
| LRRC4B | 1.0 | 1.0 | Shared | Cellular adhesion via plasma membrane adhesion molecules; Cell junction assembly | 15; 16; 17 |
| **M6PR** | 0.34 | 1.0 | Specific to LGALS8.Short | --- | --- |
| MFAP3 | 0.96 | 1.0 | Specific to LGALS8.Short | --- | 15; 16; 17 |
| NCSTN | 0.34 | 1.0 | Specific to LGALS8.Short | Cell recognition; Regulation of synaptic transmission; Cognitive activity | --- |
| NELFCD | 0.65 | 0.96 | Specific to LGALS8.Short | --- | --- |
| NPTN | 0.97 | 1.0 | Shared | Regulation of synaptic transmission; Dendrite self-avoidance; Cognitive activity; Cellular adhesion via plasma membrane adhesion molecules; Cell junction assembly; Neuron recognition | 15 |
| PTGFRN | 0.0 | 1.0 | Specific to LGALS8.Short | --- | 10; 15; 16; 17 |
| **PTPRA** | 0.96 | 0.97 | Shared | Cellular response to peptide; Activities mediated by integrin; Cell junction assembly | 15; 16; 17 |
| SLC12A9 | 0.96 | 1.0 | Shared | Regulation of synaptic transmission; Import into the cell across plasma membrane | 15; 16; 17 |
| **SLC17A5** | 0.0 | 0.95 | Specific to LGALS8.Short | Organic molecule transport | 15; 16; 17 |
| **SLC1A5** | 1.0 | 0.99 | Shared | Vascular process in circulatory system; Transport across blood-brain barrier; Organic molecule transport; Virus host interaction; Import into the cell across plasma membrane; Symbiont host interaction; Vascular transport | --- |

Gene in bold are related to osteoclast biology

**Supplemental Table S1. Continued**

| **Prey** | **SAINTexpress Score** | | **Interactor** | **GO: BP** | **References** |
| --- | --- | --- | --- | --- | --- |
|  | **LGALS8L** | **LGALS8S** |  |  |  |
| **SLC39A14** | 0.93 | 0.84 | Shared | Cellular response to peptide; Vascular transport; Import into the cell across plasma membrane | --- |
| SUSD5 | 1.0 | 1.0 | Shared | --- | 15; 16; 17 |
| TPBG | 0.0 | 0.99 | Specific to LGALS8.Short | Cognitive activity; Cell junction assembly | --- |

Gene in bold are related to osteoclast biology

All proteins interacting with galectin-8 isoforms and selected in our analysis according to the interacting scores determined by SAINTexpress (at least 0.9 to validate an interaction with one isoform, and greater than 0.7 with the other isoform to qualify common partners) have been represented, indicating the SAINTexpress score for each isoform, the type of interaction (shared or specific), and detailing the associated metabolic pathways (GO: BP). References used in this table are listed below.

**References**

1. Bell, S.L., Lopez, K.L., Cox, J.S., Patrick, K.L., Watson, R.O., 2021. Galectin-8 Senses Phagosomal Damage and Recruits Selective Autophagy Adapter TAX1BP1 To Control Mycobacterium tuberculosis Infection in Macrophages. mBio 12, e01871-20. https://doi.org/10.1128/mBio.01871-20

2. Boura-Halfon, S., Voliovitch, H., Feinstein, R., Paz, K., Zick, Y., 2003. Extracellular Matrix Proteins Modulate Endocytosis of the Insulin Receptor *. J. Biol. Chem. 278, 16397–16404. https://doi.org/10.1074/jbc.M212385200

3. Cárcamo, C., Pardo, E., Oyanadel, C., Bravo-Zehnder, M., Bull, P., Cáceres, M., Martínez, J., Massardo, L., Jacobelli, S., González, A., Soza, A., 2006. Galectin-8 binds specific β1 integrins and induces polarized spreading highlighted by asymmetric lamellipodia in Jurkat T cells. Exp. Cell Res. 312, 374–386. https://doi.org/10.1016/j.yexcr.2005.10.025

4. Chen, W.-S., Cao, Z., Sugaya, S., Lopez, M.J., Sendra, V.G., Laver, N., Leffler, H., Nilsson, U.J., Fu, J., Song, J., Xia, L., Hamrah, P., Panjwani, N., 2016. Pathological lymphangiogenesis is modulated by galectin-8-dependent crosstalk between podoplanin and integrin-associated VEGFR-3. Nat. Commun. 7, 11302. https://doi.org/10.1038/ncomms11302

5. Diskin, S., Cao, Z., Leffler, H., Panjwani, N., 2009. The role of integrin glycosylation in galectin-8-mediated trabecular meshwork cell adhesion and spreading. Glycobiology 19, 29–37. <https://doi.org/10.1093/glycob/cwn100>

6. Diskin, S., Chen, W.-S., Cao, Z., Gyawali, S., Gong, H., Soza, A., González, A., Panjwani, N., 2012. Galectin-8 Promotes Cytoskeletal Rearrangement in Trabecular Meshwork Cells through Activation of Rho Signaling. PLoS ONE 7, e44400. https://doi.org/10.1371/journal.pone.0044400

7. Hadari, Y.R., Arbel-Goren, R., Levy, Y., Amsterdam, A., Alon, R., Zakut, R., Zick, Y., 2000. Galectin-8 binding to integrins inhibits cell adhesion and induces apoptosis. J. Cell Sci. 113, 2385–2397. https://doi.org/10.1242/jcs.113.13.2385

8. Jia, J., Abudu, Y.P., Claude-Taupin, A., Gu, Y., Kumar, S., Choi, S.W., Peters, R., Mudd, M.H., Allers, L., Salemi, M., Phinney, B., Johansen, T., Deretic, V., 2018. Galectins Control mTOR in Response to Endomembrane Damage. Mol. Cell 70, 120-135.e8. https://doi.org/10.1016/j.molcel.2018.03.009

9. Levy, Y., Arbel-Goren, R., Hadari, Y.R., Eshhar, S., Ronen, D., Elhanany, E., Geiger, B., Zick, Y., 2001. Galectin-8 Functions as a Matricellular Modulator of Cell Adhesion *. J. Biol. Chem. 276, 31285–31295. https://doi.org/10.1074/jbc.M100340200

10. Lo, Y.-H., Li, C.-S., Chen, H.-L., Chiang, C.-Y., Huang, C.-C., Tu, T.-J., Lo, T.-H., Choy, D.F., Arron, J.R., Chen, H.-Y., Liu, F.-T., 2021. Galectin-8 Is Upregulated in Keratinocytes by IL-17A and Promotes Proliferation by Regulating Mitosis in Psoriasis. J. Invest. Dermatol. 141, 503-511.e9. https://doi.org/10.1016/j.jid.2020.07.021

11. Pardo, E., Barake, F., Godoy, J.A., Oyanadel, C., Espinoza, S., Metz, C., Retamal, C., Massardo, L., Tapia-Rojas, C., Inestrosa, N.C., Soza, A., González, A., 2019. GALECTIN-8 Is a Neuroprotective Factor in the Brain that Can Be Neutralized by Human Autoantibodies. Mol. Neurobiol. 56, 7774–7788. https://doi.org/10.1007/s12035-019-1621-3

12. Shatz-Azoulay, H., Vinik, Y., Isaac, R., Kohler, U., Lev, S., Zick, Y., 2020. The Animal Lectin Galectin-8 Promotes Cytokine Expression and Metastatic Tumor Growth in Mice. Sci. Rep. 10, 7375. https://doi.org/10.1038/s41598-020-64371-z

13. Yamamoto, H., Nishi, N., Shoji, H., Itoh, A., Lu, L.-H., Hirashima, M., Nakamura, T., 2008. Induction of Cell Adhesion by Galectin-8 and its Target Molecules in Jurkat T-Cells. J. Biochem. (Tokyo) 143, 311–324. https://doi.org/10.1093/jb/mvm223

14. Zamorano, P., Koning, T., Oyanadel, C., Mardones, G.A., Ehrenfeld, P., Boric, M.P., González, A., Soza, A., Sánchez, F.A., 2019. Galectin-8 induces endothelial hyperpermeability through the eNOS pathway involving S-nitrosylation-mediated adherens junction disassembly. Carcinogenesis 40, 313–323. <https://doi.org/10.1093/carcin/bgz002>

Database

15. Oughtred, R., Stark, C., Breitkreutz, B.-J., Rust, J., Boucher, L., Chang, C., Kolas, N., O’Donnell, L., Leung, G., McAdam, R., Zhang, F., Dolma, S., Willems, A., Coulombe-Huntington, J., Chatr-aryamontri, A., Dolinski, K., Tyers, M., 2019. The BioGRID interaction database: 2019 update. Nucleic Acids Res 47, D529–D541. <https://doi.org/10.1093/nar/gky1079>

16. Huttlin, E.L., Bruckner, R.J., Navarrete-Perea, J., Cannon, J.R., Baltier, K., Gebreab, F., Gygi, M.P., Thornock, A., Zarraga, G., Tam, S., Szpyt, J., Gassaway, B.M., Panov, A., Parzen, H., Fu, S., Golbazi, A., Maenpaa, E., Stricker, K., Thakurta, S.G., Zhang, T., Rad, R., Pan, J., Nusinow, D.P., Paulo, J.A., Schweppe, D.K., Vaites, L.P., Harper, J.W., Gygi, S.P., 2021. Dual Proteome-scale Networks Reveal Cell-specific Remodeling of the Human Interactome. Cell 184, 3022-3040.e28. <https://doi.org/10.1016/j.cell.2021.04.011>

17. Del Toro, N., Shrivastava, A., Ragueneau, E., Meldal, B., Combe, C., Barrera, E., Perfetto, L., How, K., Ratan, P., Shirodkar, G., Lu, O., Mészáros, B., Watkins, X., Pundir, S., Licata, L., Iannuccelli, M., Pellegrini, M., Martin, M.J., Panni, S., Duesbury, M., Vallet, S.D., Rappsilber, J., Ricard-Blum, S., Cesareni, G., Salwinski, L., Orchard, S., Porras, P., Panneerselvam, K., Hermjakob, H., 2021. The IntAct database: efficient access to fine-grained molecular interaction data. Nucleic Acids Res 50, D648–D653. <https://doi.org/10.1093/nar/gkab1006>
