# Supplementary material for: Galectin-8 modulates human osteoclast activity partly through isoform-specific interactions
Source: Life Sci Alliance. 2024 Feb 23;7(5):e202302348. doi: 10.26508/lsa.202302348 (PMC10895193; doi:10.26508/lsa.202302348)
Supplement: Supplementary file 2 [file LSA-2023-02348_TableS2.docx]

**Supplemental Table S2.** Psm of peptides from N-CRD/C-CRD domains of both isoforms

This table shows the number of psm (peptide spectrum matches), i.e. the number of occurrences of each peptide in the N-CRD and C-CRD domains. N-CRD and C-CRD scores represent average levels of CRD domain abundance, while isoform-specific ratios provide information on the abundance of one CRD relative to another. Thus, a low or high N-CRD/C-CRD ratio would indicate that one functional domain is more abundant and the other less abundant (due to a likely cleavage process) and therefore unable to promote an interaction, whereas a ratio of 1 would indicate that both domains are proportionally involved in establishing the interaction. *NA: Not available; psm: peptide-spectrum match*

|  | **Samples LGALS8S** | |  | **Samples LGALS8L** | |  |
| --- | --- | --- | --- | --- | --- | --- |
|  | **psm LGALS8S N1 SPC** | **psm LGALS8S N3 SPC** |  | **psm**  **LGALS8L**  **N1 SPC** | **psm**  **LGALS8L**  **N3 SPC** |  |
| N-CRD peptides [18 peptides] |  |  |  |  |  |  |
|  |  |  | mean |  |  | mean |
| ADVAFHFNPR | 2 | 2 | 2 | 2 | 2 | 2 |
| AGCIVCNTLINEK | 2 | 2 | 2 | 2 | 2 | 2 |
| DKFQVAVNGK | 1 | 1 | 1 | 1 | 1 | 1 |
| DKFQVAVNGKHTLLYGHR | 1 | 1 | 1 | NA | NA | NA |
| EEITYDTPFK | 1 | 1 | 1 | 1 | 1 | 1 |
| EEITYDTPFKR | 2 | 3 | 2.5 | 2 | 2 | 2 |
| EKSFEIVIMVLK | 3 | 4 | 3.5 | 2 | 4 | 3 |
| EKSFEIVIMVLKDK | 2 | 2 | 2 | 2 | 2 | 2 |
| FQVAVNGK | 1 | 1 | 1 | 1 | 1 | 1 |
| HTLLYGHR | 3 | 4 | 3.5 | 4 | 2 | 3 |
| IDTLGIYGK | 3 | 3 | 3 | 2 | 3 | 2.5 |
| IGPEKIDTLGIYGK | 2 | 3 | 2.5 | 3 | 2 | 2.5 |
| RAGCIVCNTLINEK | 4 | 3 | 3.5 | 2 | 3 | 2.5 |
| SFEIVIMVLK | 2 | 2 | 2 | 2 | 2 | 2 |
| SFEIVIMVLKDK | 1 | 1 | 1 | 1 | 1 | 1 |
| SFEIVIMVLKDKFQVAVNGK | 2 | 2 | 2 | 2 | 2 | 2 |
| WGREEITYDTPFK | 2 | 3 | 2.5 | 2 | 2 | 2 |
| WGREEITYDTPFKR | 7 | 6 | 6.5 | 4 | 4 | 4 |
|  | N-CRD score | | 2.3611 | N-CRD score | | 2.0882 |
| C-CRD peptides [16 peptides] |  |  |  |  |  |  |
|  |  |  | mean |  |  | mean |
| DIALHLNPR | 4 | 2 | 3 | 3 | 2 | 2.5 |
| EFKVAVNGVHSLEYK | 1 | 1 | 1 | NA | 1 | 1 |
| ELSSIDTLEINGDIHLLEVR | 2 | 3 | 2.5 | 2 | 2 | 2 |
| ELSSIDTLEINGDIHLLEVRSW | 1 | 1 | 1 | NA | 1 | 1 |
| FKELSSIDTLEINGDIHLLEVR | 33 | 32 | 32.5 | 13 | 24 | 18.5 |
| GEVNANAKSFNVDLLAGK | 2 | 2 | 2 | 2 | 2 | 2 |
| LNIKAFVR | 2 | 2 | 2 | 2 | 2 | 2 |
| LNTPMGPGR | 2 | 4 | 3 | 3 | 2 | 2.5 |
| LNTPMGPGRTVVVKGEVNANAK | 1 | NA | 1 | NA | NA | NA |
| NITSFPFSPGMYFEMIIYCDVR | 7 | 7 | 7 | 5 | 6 | 5.5 |
| NSFLQESWGEEER | 5 | 6 | 5.5 | 4 | 4 | 4 |
| SFNVDLLAGK | 4 | 3 | 3.5 | 2 | 3 | 2.5 |
| SFNVDLLAGKSK | 1 | 1 | 1 | 1 | 2 | 1.5 |
| SKDIALHLNPR | 2 | 3 | 2.5 | 2 | 3 | 2.5 |
| TVVVKGEVNANAK | 5 | 3 | 4 | 3 | 4 | 3.5 |
| VAVNGVHSLEYK | 2 | 2 | 2 | 2 | 2 | 2 |
|  | C-CRD score | | 4.5937 | C-CRD score | | 3.5333 |
| ratio 1: N-CRD short score / C-CRD short score = 0.514 | | | | | | |
| ratio 2: N-CRD long score / C-CRD long score = 0.591 | | | | | | |
