# Supplementary material for: Galectin-8 modulates human osteoclast activity partly through isoform-specific interactions
Source: Life Sci Alliance. 2024 Feb 23;7(5):e202302348. doi: 10.26508/lsa.202302348 (PMC10895193; doi:10.26508/lsa.202302348)
Supplement: Supplementary file 3 [file LSA-2023-02348_TableS3.docx]

**Supplemental Table S3.** Grouping of GO terms

| **MF: Molecular Function** |
| --- |
| - *Transmembrane transporter activity:* |
| GO:0022804 (active transmembrane transporter activity), |
| GO:0022853 (active ion transmembrane transporter activity), |
| GO:0008509 (anion transmembrane transporter activity), |
| GO:0140359 (ABC-type transporter activity), |
| GO:0015291 (secondary active transmembrane transporter activity), |
| GO:0015293 (symporter activity), |
| GO:0042626 (ATPase-coupled transmembrane transporter activity), |
| GO:0015399 (primary active transmembrane transporter activity), |
| GO:0015562 (efflux transmembrane transporter activity), |
| GO:0015296 (anion:cation symporter activity), |
| GO:0090482 (vitamin transmembrane transporter activity), |
| GO:0042910 (xenobiotic transmembrane transporter activity). |
|  |
| **KEGG_Terms_ID** |
| - *Cardiomyopathy:* |
| hsa05412 (Arrhythmogenic right ventricular cardiomyopathy (ARVC)), |
| hsa05410 (Hypertrophic cardiomyopathy (HCM)), |
| hsa05414 (Dilated cardiomyopathy). |
|  |
| **BP: Biological Process** |
| - *Symbiont host interaction:* |
| GO:0044409 (entry into host), |
| GO:0052126 (movement in host environment), |
| GO:0051701 (biological process involved in interaction with host); |
| - *Import into the cell across plasma membrane*: |
| GO:0098657 (import into cell), |
| GO:0098739 (import across plasma membrane); |
| - *Cognitive activity:* |
| GO:0007611 (learning or memory), |
| GO:0050890 (cognition), |
| GO:0008306 (associative learning); |
| - *Response to amyloid-beta:* |
| GO:1904646 (cellular response to amyloid-beta), |
| GO:1904645 (response to amyloid-beta); |
| - *Organic molecule transport:* |
| GO:0046942 (carboxylic acid transport), |
| GO:0015711 (organic anion transport); |
| - *Regulation of synaptic transmission:* |
| GO:1900271 (regulation of long-term synaptic potentiation), |
| GO:0060291 (long-term synaptic potentiation), |
| GO:0050804 (modulation of chemical synaptic transmission), |
| GO:0099177 (regulation of trans-synaptic signaling); |
| - *Activities mediated by integrin:* |
| GO:0033631 (cell-cell adhesion mediated by integrin), |
| GO:0007229 (integrin-mediated signaling pathway), |
| GO:0033627 (cell adhesion mediated by integrin); |
| - *Virus host interaction:* |
| GO:0046718 (viral entry into host cell), |
| GO:0019058 (viral life cycle); |
| - *Cellular adhesion via plasma membrane adhesion molecules:* |
| GO:0098742 (cell-cell adhesion via plasma-membrane adhesion molecules), |
| GO:0007156 (homophilic cell adhesion via plasma membrane adhesion molecules); |
| - *Organization of extracellular structure components:* |
| GO:0030198 (extracellular matrix organization), |
| GO:0043062 (extracellular matrix organization), |
| GO:0045229 (external encapsulating structure organization); |
| - *Cell junction assembly:* |
| GO:0034329 (cell junction assembly), |
| GO:190188 (regulation of cell junction assembly) |
|  |
