# Supplementary material for: Galectin-8 modulates human osteoclast activity partly through isoform-specific interactions
Source: Life Sci Alliance. 2024 Feb 23;7(5):e202302348. doi: 10.26508/lsa.202302348 (PMC10895193; doi:10.26508/lsa.202302348)
Supplement: Supplementary file 4 [file LSA-2023-02348_TableS4.docx]

**Supplemental Table S4.** Immunoblot antibodies information

| **Antigen** | **Catalog #** | **Host** | **Clonality** | **Supplier** | **Dilution** |
| --- | --- | --- | --- | --- | --- |
| Galectin-8 | sc-377133 | Mouse | Monoclonal | Santa Cruz Biotechnology | 1/1000 |
| Actin | A2066 | Rabbit | Polyclonal | Sigma-Aldrich (Millipore Sigma) | 1/4000 |
| Phospho-ULK1  Ser^757^ | 6888 | Rabbit | Polyclonal | Cell Signaling Technology | 1/1000 |
| ULK1 | 8054 | Rabbit | Monoclonal | Cell Signaling Technology | 1/1000 |
| Phospho-p70 S6 Kinase Thr^229^ | 44-918G | Rabbit | Polyclonal | Invitrogen (ThermoFisher Scientific) | 1/1000 |
| p70 S6 Kinase | 2708 | Rabbit | Monoclonal | Cell Signaling Technology | 1/1000 |
| LC3B | 2775 | Rabbit | Polyclonal | Cell Signaling Technology | 1/1000 |
| Phospho-4E-BP1  Thr^37/46^ | 2855 | Rabbit | Monoclonal | Cell Signaling Technology | 1/1000 |
| Non-Phospho-4E-BP1 Thr^46^ | 4923 | Rabbit | Monoclonal | Cell Signaling Technology | 1/1000 |
| Phospho-Raptor  Ser^792^ | 2083 | Rabbit | Polyclonal | Cell Signaling Technology | 1/1000 |
| Raptor | 2280 | Rabbit | Monoclonal | Cell Signaling Technology | 1/1000 |
| Phospho-PDK1  Ser^241^ | 3438 | Rabbit | Monoclonal | Cell Signaling Technology | 1/1000 |
| PDK1 | 5662 | Rabbit | Monoclonal | Cell Signaling Technology | 1/1000 |
| Phospho-AMPKα  Thr^172^ | 2535 | Rabbit | Monoclonal | Cell Signaling Technology | 1/1000 |
| AMPKα | 5832 | Rabbit | Monoclonal | Cell Signaling Technology | 1/1000 |
| Phospho-Akt  Ser^473^ | 4060 | Rabbit | Monoclonal | Cell Signaling Technology | 1/1000 |
| Akt | 4691 | Rabbit | Monoclonal | Cell Signaling Technology | 1/1000 |
| Phospho-Erk1/2  Thr^202^/Tyr^204^ | 4370 | Rabbit | Monoclonal | Cell Signaling Technology | 1/2000 |
| Erk1/2 | 4695 | Rabbit | Monoclonal | Cell Signaling Technology | 1/2000 |
| Flag epitope | F7425 | Rabbit | Polyclonal | Millipore (Millipore Sigma) | 1/2000 |
