# Supplementary material for: Galectin-8 modulates human osteoclast activity partly through isoform-specific interactions
Source: Life Sci Alliance. 2024 Feb 23;7(5):e202302348. doi: 10.26508/lsa.202302348 (PMC10895193; doi:10.26508/lsa.202302348)
Supplement: Supplementary file 5 [file LSA-2023-02348_TableS5.docx]

**Supplemental Table S5.** Immunofluorescence antibodies information

| **Antigen** | **Catalog #** | **Host** | **Clonality** | **Supplier** | **Dilution** |
| --- | --- | --- | --- | --- | --- |
| Galectin-8 | HPA012734 | Rabbit | Polyclonal | Sigma-Aldrich (Millipore Sigma) | 1/50 |
| Galectin-8 | MAB13051 | Mouse | Monoclonal | R&D Systems (Bio-techne) | 1/50 |
| Alexa Fluor^TM^ 488 Phalloidin | A12379 | --- | --- | Invitrogen (ThermoFisher Scientific) | 1/150 |
| CLCN3 | 13359S | Rabbit | Monoclonal | Cell Signaling Technology | 1/50 |
| CLCN7 | HPA043019 | Rabbit | Polyclonal | Sigma-Aldrich (Millipore Sigma) | 1/100 |
| LAMP1 | sc-18821 | Mouse | Monoclonal | Santa Cruz Biotechnology | 1/50 |
| LAMP2 | sc-18822 | Mouse | Monoclonal | Santa Cruz Biotechnology | 1/100 |
