# Supplementary material for: Galectin-8 modulates human osteoclast activity partly through isoform-specific interactions
Source: Life Sci Alliance. 2024 Feb 23;7(5):e202302348. doi: 10.26508/lsa.202302348 (PMC10895193; doi:10.26508/lsa.202302348)
Supplement: Supplementary file 6 [file LSA-2023-02348_TableS6.docx]

**Supplemental Table S6.** Sequences of small interfering RNAs (DsiRNA, siRNA)

|  | **Sequences** | **Nucleotides** |
| --- | --- | --- |
| Galectin-8  DsiRNA #1 | 5’- GUC CUU AAA CAA CCU ACA GAA UAT C - 3’  3’ - AAC AGG AAU UUG UUG GAU GUC UUA UAG - 5’ | 231-256 |
| Galectin-8  DsiRNA #2 | 5’- CGC CUG AAU AUU AAA GCA UUU GUA A - 3’  3’-GUG CGG ACU UAU AAU UUC GUA AAC AUU - 5’ | 919-944 (Short isoform CDS)  1045-1070 (Long isoform CDS) |
| Negative control DsiRNA | 5’- CGU UAA UCG CGU AUA CGC GUA T -3’  3’- CAG CAA UUA GCG CAU AUU AUG CGC AUA -5’ | --- |
| Long Galectin-8  siRNA | 5’- UCU UGG UGU AGA CAG UUC UGG - 3’  3’- CG AGA ACC ACA UCU GUC AAG A - 5’ | 586-609 (Long isoform CDS) |
| Short Galectin-8  siRNA | 5’- UGG CAG CCU AAG CUG GGG CGU - 3’  3’- UU ACC GUC GGAU UCG ACC CCG – 5’ | 537-560 (Short isoform CDS) |
| AllStars Negative control siRNA | Sequence not provided by the manufacturer  (Qiagen catalog # 1027280) | --- |
